# Chromium [Cr(VI)] biosorption property of the newly isolated actinobacterial probiont *Streptomyces werraensis* LD22

**DOI:** 10.1007/s13205-014-0237-6

**Published:** 2014-07-14

**Authors:** S. Latha, G. Vinothini, D. Dhanasekaran

**Affiliations:** Bioprocess Technology Laboratory, Department of Microbiology, School of Life Sciences, Bharathidasan University, Tiruchirappalli, 620 024 Tamil Nadu India

**Keywords:** Heavy metals, Actinobacteria, Probiotics, Metal biosorbent

## Abstract

The present work demonstrates the heavy metal resistance and detoxification of Cr(VI) by the probiotic actinobacterial cultures isolated from chicken and goat feces. The actinobacterial isolates were screened for heavy metal resistance by qualitative, semiquantitative assays and Cr(VI) biosorption was determined by analytical techniques such as atomic absorption spectrophotometry and Fourier transform infrared spectrometry (FT-IR). All the tested actinobacterial isolates (*n* = 20) showed resistance toward K_2_Cr_2_O_7_, NiCl_2_, ZnCl_2_, CuSO_4_ and PbNO_3_ at 20 mg L^−1^ concentration. The maximum tolerance concentration values were found to be 200–250 mg L^−1^ for K_2_Cr_2_O_7_, 100–250 mg L^−1^ for PbNO_3_ and <50–250 mg L^−1^ for NiCl_2_, ZnCl_2_ and CuSO_4_. Among the five tested heavy metals, Cr(VI) was resisted by 95 % of the tested actinobacterial cultures up to 250 mg L^−1^ concentration; particularly, the isolate LD22 exhibited a high degree of tolerance to all the tested heavy metals. Thus, the isolate was justifiably chosen for Cr(VI) biosorption study and the biosorption efficacy was found maximum at 100 mg L^−1^ of metal ion concentration (3 g L^−1^ of biomass dosage and pH 7.0). FT-IR spectrum revealed the chemical interactions between the hydroxyl, amine and carboxyl groups of the biomass and the metal ions. On the basis of phenotypic, physiological, biochemical and molecular characteristics the isolate LD22 was identified as *Streptomyces werraensis* LD22 (JX524481) which could be used to develop a biosorbent for adsorbing Cr(VI) metal ions.

**Electronic supplementary material:**

The online version of this article (doi:10.1007/s13205-014-0237-6) contains supplementary material, which is available to authorized users.

## Introduction

Heavy metals are widespread pollutants of great concern as they are non-biodegradable and thus persistent in nature. They occur naturally in the environment, but their concentrations are frequently elevated as a result of pollution introduced into the environment through different human activities such as mining, smelting, sewage sludge disposal, application of pesticides, inorganic fertilizers and atmospheric emissions (Liu et al. 2005). Heavy metal contamination is a serious problem not only from the environmental viewpoint, but also from human and animal health perspective because of its non-biodegradable, hazardous and toxic properties. All living organisms require varying amounts of some of these heavy metals for their life processes. Certain mineral elements like iron, manganese and zinc are essential dietary nutrients for poultry and livestock. However, all mineral elements, whether considered to be essential or potentially toxic, can have an adverse effect upon humans and animals if included in the diet at excessively high concentrations (Okoye et al. 2011). Humans and animals have also been associated with anthropogenic heavy metal pollution in many other ways such as consumption of metal contaminated drinking water, exposure to contaminated soils or industrial waste, absorption through the skin and inhalation of air pollutants (Tataruch 1995).

Typically in livestock, the metal ions that enter by means of the above processes are absorbed through the gastrointestinal tract into the bloodstream and reach different parts of the body where they disturb essential metabolic functions. Animal health impacts due to heavy metals range from enzyme inhibition, immune system degradation, neuron signal interference, organ specific degradation, reduction in fitness, reproductive interference to carcinoma, and with many exposures being lethal (Merrill et al. 2007; Hogan 2011). Moreover, heavy metals in high concentrations also affect the gut microflora of the animals by increasing intracellular concentration with the consequence of inhibition of enzymes or DNA damage by the production of reactive oxygen species or irreversible binding to the active centers of enzymes (Lopez-Maury et al. 2002). A variety of conventional physical and chemical methods are available for the removal of heavy metals from the environment. However, they are expensive, not eco-friendly and usually dependent on the concentration of metal ions. As a result, the use of microbes has emerged as an option for developing economic and eco-friendly processes for the removal of hazardous heavy metals.

Extensive research has shown that different bacteria are tolerant to heavy metals and play important roles in mobilization of heavy metals (Idris et al. 2004). Different microorganisms such as bacteria, fungi, yeast and actinobacteria, chiefly of the genus *Streptomyces*, have been tested for their biosorption potential of hazardous heavy metals. Recently, application of favorable microorganisms as probiotic is a potentially emerging field for the welfare of humans and animals. Generally, the probiotic microbes such as lactic acid bacteria (LAB) and *Bifidobacteria* demonstrated positive health effects on humans and animals with respect to nutritional, growth, disease controlling and immunological aspects. Besides probiotic properties, LAB strains also have the ability to remove cyanotoxins from water, mycotoxins from food (Meriluoto et al. 2005; Nybom et al. 2007) and to absorb aflatoxin B1 from the gastrointestinal tract of humans (Pierides et al. 2000; El-Nezami et al. 2006). But no attempt has been made so far to isolate the metal-removing actinobacterial probiont from the animal gut system through assessing the probiotic, metal-resistant and metal-removal characteristics. Thus, the present study was aimed to evaluate the heavy metal resistance and removal properties of actinobacterial probionts isolated from chicken and goat feces.

## Methods

### Maintenance and culturing conditions of actinobacterial isolates

The actinobacterial isolates (*n* = 20) used in this study were isolated from the fecal samples of chicken and goat collected from various locations in Pudukkottai and Tiruchirappalli Districts, Tamil Nadu, India (Latha and Dhanasekaran 2013). Furthermore, they have fulfilled the preface probiotic characteristics such as acid tolerance, bile tolerance, antibacterial activity, etc. (Latha et al. 2014, Personal communication). All the actinobacterial isolates were maintained in starch casein agar slants (g L^−1^; 10.0 soluble starch, 2.0 KNO_3_, 2.0 NaCl, 0.3 casein, 2.0 K_2_HPO_4_, 0.05 MgSO_4_·7H_2_O, 0.02 CaCO_3_, 0.01 FeSO_4_·7H_2_O, 15.0 agar, pH 7.2) at 4 ± 2 °C and subcultured twice prior to the experiments.

### Evaluation of actinobacterial isolates for metal resistance profiles

#### Preparation of metal solutions

The heavy metals used for the screening of metal resistance in actinobacterial isolates were chromium (Cr), mercury (Hg), lead (Pb), nickel (Ni), zinc (Zn) and copper (Cu). The salt solutions were prepared from analytical-grade chemicals such as K_2_Cr_2_O_7_, HgCl_2_, PbNO_3_, NiCl_2_, ZnCl_2_, CuSO_4_ and sterilized separately for 15 min at 110 °C (Saurav and Kannabiran 2009). The concentrations of all metal ions were prepared from stock solutions of 1,000 mg L^−1^. Fresh dilutions were used for each study.

#### Primary qualitative assay

The probiotic actinobacterial isolates were screened for heavy metal-resistant activity in minimal medium agar (MMA, g L^−1^; 10.0 glucose, 0.5 L-asparagine, 0.5 K_2_HPO_4_, 0.2 MgSO_4_·7H_2_O, 0.01 FeSO_4_·7H_2_O, 15.0 agar). The MMA medium was prepared and sterilized by autoclaving at 121 °C and 15 lbs for 15 min. After sterilization, 20 mg L^−1^ concentration of each sterile metal salt solution was supplemented independently into the MMA medium before solidification. Then the test actinobacterial isolates were inoculated and incubated at 41 ± 2 °C for 7 days. A control plate not supplemented with the metals was maintained to assess the normal growth of actinobacteria (Koushalshahi et al. 2012).

#### Secondary semiquantitative assay

The maximum tolerance concentration (MTC) for the selected actinobacterial cultures against the heavy metals was determined by semiquantitative well diffusion assay according to the method of Sanjenbam et al. (2012) with slight modifications. Stationary phase probiotic actinobacterial cultures were uniformly spread using sterile cotton swab on plates containing MMA medium. Then, wells of 10 mm diameter were cut out using a sterile cork borer. Five serial dilutions yielding concentrations of 50, 100, 150, 200 and 250 mg L^−1^ for PbNO_3_, K_2_Cr_2_O_7_, NiCl_2_, ZnCl_2_ and CuSO_4_ were prepared. Fifty microliters of each concentration was added to each well of the MMA medium inoculated with the test actinobacterial cultures. The plates were incubated at 41 ± 2 °C for 7 days and examined for the presence of the zone of inhibition around the wells. Inhibition of the actinobacterial growth was measured in millimeters. The cultures showing inhibition zones of <7 and >10 mm were considered as resistant and sensitive, respectively, for the metals according to the earlier reports of Koushalshahi et al. (2012) and Yadav et al. (2009). The maximum concentration of metals in which the actinobacterial growth present was found to be positive and have been recorded as the MTC value of that particular actinobacterial culture.

#### Biomass preparation of actinobacterial biosorbent

The biomass of actinobacterial biosorbent was prepared according to the method of Saurav and Kannabiran (2011a) with slight modifications. The actinobacterial isolate LD22 was cultivated in 500 mL Erlenmeyer flasks containing 200 mL of starch casein broth and kept shaking in an orbital rotary shaker at 100 rpm for 15 days at 41 ± 2 °C. Then the culture was harvested by centrifugation at 13,000 rpm for 5 min and washed three times with distilled water. The pellet was kept in petri dishes and dried at 70 °C for 24 h. Then the dried biomass was used for further analysis.

#### Biosorption of Cr(VI)

The chromium removal ability of the actinobacterial isolate LD22 was determined by measuring the level of chromium uptake following the method of Saurav and Kannabiran (2011b) with slight modifications. In the stoppered conical flasks (100 mL), 25 mL of Cr(VI) metal salt solution (pH 7.0) in the concentration of 50–250 mg L^−1^ was prepared and resuspended with 3.0 g L^−1^ dried biomass of actinobacterial isolate LD22. Then the flasks were kept shaking in an orbital rotary shaker at 100 rpm for 1 week. The contents of the flasks were filtered through Whatman no.1 filter paper and the filtrates analyzed for chromium concentration by flame atomic absorption spectrophotometer (AAS, 400/HGA 900/AS 800-Perkin Elmer). The metal removal efficiency (MRE) was calculated by using the following equation:

$$ {\text{MRE }}\% = \left[ {\left( {C_{\text{i}} - \, C_{\text{f}} /C_{\text{i}} \, \times \, 100} \right)} \right] $$

where *C*_i_ represents the initial chromium metal ion concentration and *C*_f_ represents the final chromium metal ion concentration.

#### FT-IR analysis

To determine the changes in surface characteristics (conformational changes in functional groups) of the cells grown in the presence of Cr(VI), the infrared spectra of the normal and Cr(VI)-treated LD22 actinobacterial cells were studied using Fourier transform infrared spectrometer (Spectrum Two-Perkin Elmer) according to the protocol of Bhattacharya and Gupta (2013) with minor modification. To carry out the analysis, conical flasks containing 25 mL of minimal medium broth supplemented with and without 100 mg L^−1^ of Cr(VI) were inoculated with 3.0 g L^−1^ dried biomass of actinobacterial isolate LD22. The flasks were kept shaking in an orbital rotary shaker at 100 rpm for 7 days and centrifuged at 9,500 rpm and 4 °C for 10 min to pellet down the cell mass. The pellets were washed with saline prior to drying overnight at 60 °C. The dried pellets were then crushed to fine powder and the resultant biomass was mixed with KBr in the ratio of about 1:100 to form KBr disks. The FT-IR spectra of dried biomass in the KBr phase was recorded using FT-IR spectrometer in the range of 400–4,000 cm^−1^.

### Taxonomic identification of the heavy metal-resistant probiont LD22

#### Phenotypic characterization

Morphological features of the heavy metal-resistant probiont LD22 were studied by Gram staining and coverslip culture technique (Augustine et al. 2005; Dhanasekaran et al. 2009; Dhanasekaran 2010) using light, phase contrast and scanning electron microscope. The cultural characteristics such as color of aerial, substrate mycelium, growth pattern, colony size and consistency were monitored on 7, 14 and 21 day-old cultures of the isolate LD22. These characteristics were tested on the basis of the observations made on International Streptomyces Project (ISP) media recommended by Shirling and Gottileb (1966) and Bennett agar. Melanin production was tested in ISP medium 6 (peptone-yeast extract iron agar) and 7 (tyrosine agar) (Smaoui et al. 2012).

The utilization of sole carbon and nitrogen sources by the isolate LD22 was investigated in ISP medium 9 and basal medium, respectively (Saha et al. 2013). The effects of salt and pH on the growth of the isolate LD22 was assessed by using modified Bennett agar medium supplemented with graded doses of NaCl (2, 4, 6, 8 and 10 %) and by varying the pH of the medium (5–10) with respect to the method of Shirling and Gottileb (1966). Various biochemical tests were performed for the identification of the potent isolate LD22 which includes indole test, methyl red test, voges–proskauer test, citrate utilization, carbohydrate fermentation, hydrogen sulfide production, catalase test, cytochrome oxidase test, casein hydrolysis, urea hydrolysis, starch hydrolysis, lipid hydrolysis and gelatin hydrolysis(Pridham and Gottlieb 1948; Nonomura 1974). The isolate was compared and identified according to Bergey’s manual of determinative bacteriology.

#### Genotypic characterization

The genomic DNA of the probiont LD22 was isolated by modified high salt method as described by Perry and Pasi (1999). PCR amplification of 16S rRNA gene of the isolate LD22 was performed in an automated Thermal Cycler (Applied Biosystems) using universal primers 27f (5′-AGAGTTTGATCMTGGCTCAG-3′) and 765r (5′-CTGTTTGCTCCCCACGCTTTC-3′) according to the amplification profile described by Coombs and Franco (2003). The PCR product was analyzed by agarose gel electrophoresis and DNA of the expected size was purified and then subjected to sequencing at Macrogen Inc., Korea. The 16S rRNA gene sequence obtained was searched through the NCBI database using the BLAST algorithm to identify the closest matches. The sequence was aligned with representative actinobacterial 16S rRNA gene sequences and a phylogenetic tree was constructed using the CLUSTALW and MEGA software version 5. Restriction enzyme site analysis and secondary structure prediction for 16S rRNA gene were carried out using NEBcutter program version 2.0 (http://tolls.neb.com/NEBCutter2/index.php) and GeneBee software (http://www.genebee.msu.su/services/rna2-reduced.html), respectively (Dhanasekaran et al. 2012).

## Results

### Metal resistance profiles of probiotic actinobacterial isolates

**Fig. 1:**
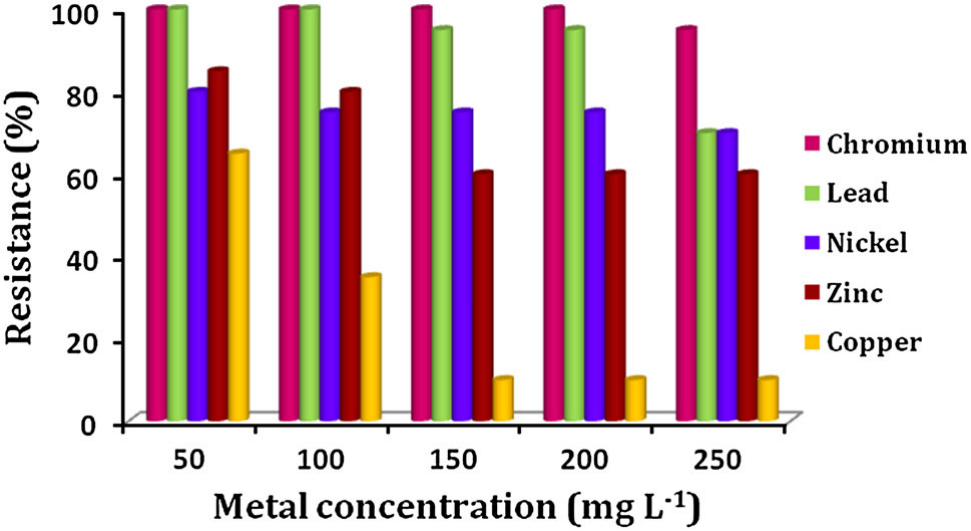
Percentage of probiotic actinobacterial isolates resistant to various concentrations of heavy metals

The actinobacterial cultures (n = 20) isolated from chicken and goat with probiotic properties (Latha et al. 2014) were checked for their resistance to hazardous heavy metals (K_2_Cr_2_O_7_, HgCl_2_, PbNO_3_, NiCl_2_, ZnCl_2_ and CuSO_4_) by primary qualitative assay. All the 20 isolates showed a clear visible growth in the minimal medium agar supplemented with the metals K_2_Cr_2_O_7_, NiCl_2_, CuSO_4_, PbNO_3_ and ZnCl_2,_ whereas no growth was observed in the HgCl_2_ supplemented plates which clearly indicated that the isolates were resistant to K_2_Cr_2_O_7_, NiCl_2_, CuSO_4_, PbNO_3_ and ZnCl_2_ and sensitive to HgCl_2_ at 20 mg L^−1^ concentration. As a result, the isolates were further evaluated for maximum tolerance concentration (MTC) of metals by semiquantitative agar well diffusion assay. Based on their growth in the preliminary screening, the metal concentration selected for the MTC assay was 50–250 mg L^−1^. The results of this experiment demonstrated that all the 20 isolates were highly tolerant to K_2_Cr_2_O_7_ at 250 mg L^−1^ concentration except LD23, which had the ability to resist up to 200 mg L^−1^. The resistance percentage of the isolates to heavy metals varied from 95 to 100 % for K_2_Cr_2_O_7_, 70 to 100 % for PbNO_3_, 70 to 80 % for NiCl_2_, 60 to 85 % for ZnCl_2_ and 10 to 65 % for CuSO_4_ (Fig. 1). The MTC values of the isolates were found to be 100–250 mg L^−1^ for PbNO_3_ and **<**50–250 mg L^−1^ for NiCl_2_, ZnCl_2_ and CuSO_4_. Among the 20 isolates, 40, 25 and 5 % of the isolates showed high MTC values of 250 mg L^−1^ for 3, 4 and 5 metals, respectively (Table 1). From this, the only isolate LD22 exhibiting a high degree of tolerance to all the tested heavy metals was selected for further biosorption studies.

**Table 1:** Maximum tolerance concentration (MTC) of probiotic actinobacterial isolates for heavy metals

S. no.	Isolate code	Maximum tolerance concentration (MTC) in mg L^−1^				
		Chromium	Lead	Nickel	Zinc	Copper
1	JD4	250	250	**<**50	**<**50	50
2	JD5	250	250	250	50	**<**50
3	JD7	250	250	**<**50	250	100
4	JD9	250	250	250	250	100
5	JD11	250	250	250	250	50
6	JD12	250	100	250	250	**<**50
7	JD13	250	200	250	**<**50	**<**50
8	JD15	250	250	250	250	**<**50
9	JD17	250	250	250	250	100
10	JD18	250	250	50	250	50
11	LD1	250	200	250	100	**<**50
12	LD3	250	200	250	250	**<**50
13	LD3A	250	250	**<**50	100	50
14	LD8	250	200	**<**50	250	50
15	LD15	250	250	250	100	100
16	LD18	250	250	200	250	**<**50
17	LD20	250	250	250	250	100
18	LD21	250	250	250	100	50
19	LD22	250	250	250	250	250
20	LD23	200	200	250	250	250

### Biosorption of Cr(VI)

Atomic absorption spectroscopy (AAS) is one of the important analytical techniques used to ascertain the concentration of metal ions in a solution. Therefore, in this study, AAS was used to measure the efficacy of Cr(VI) biosorption by the isolate LD22. The metal concentrations used in the semiquantitative assay (50–250 mg L^−1^) were chosen for Cr(VI) biosorption experiment. More than 50 % of Cr(VI) biosorption was found in all the metal concentrations at a biomass dosage of 3 g L^−1^ of the isolate LD22. The percentages of Cr(VI) biosorption at various metal concentrations were 64.9 % (50 mg L^−1^), 82.3 % (100 mg L^−1^), 73.1 % (150 mg L^−1^), 69.5 % (200 mg L^−1^) and 51.7 % (250 mg L^−1^). The Cr(VI) biosorption ability was found to be maximum at 100 mg L^−1^ metal concentration. Besides, the biosorption ability of the isolate LD22 was significantly decreased with increase in metal concentration.

**Fig. 2:**
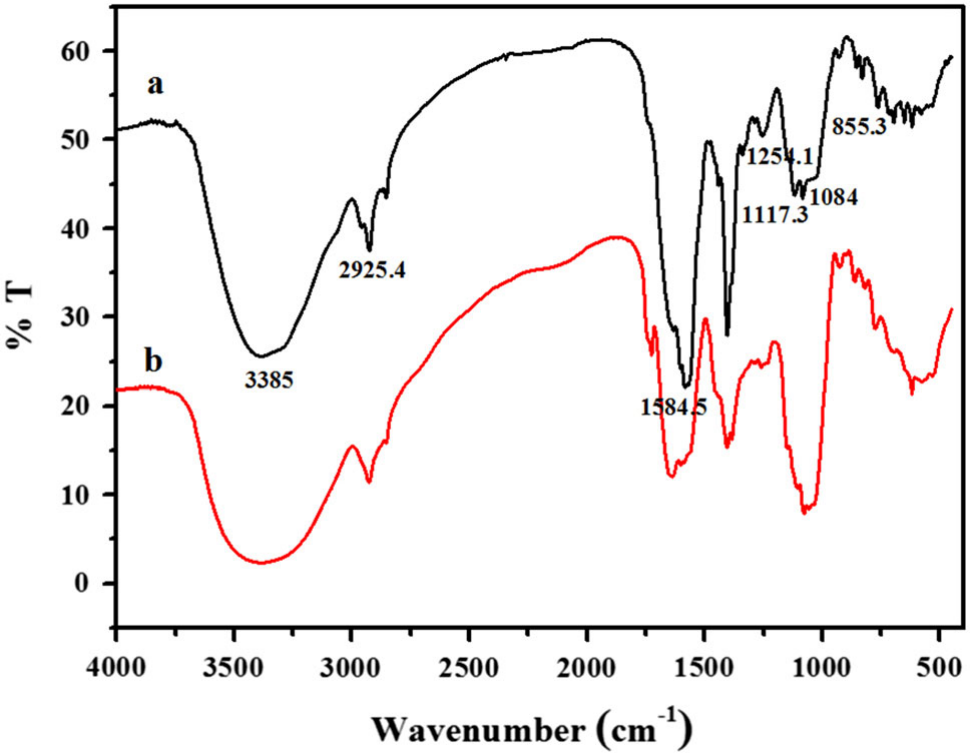
Fourier transform infrared spectra of LD22 cells grown in *a* absence and *b* presence of Cr(VI)

FT-IR analysis for Cr(VI)-treated and untreated biomass of LD22 was carried out to find the role of functional groups involved in absorption of chromium. The FT-IR spectrum of native culture biomass grown in minimal medium broth without Cr(VI) suggests the presence of a broad band at 3,385 cm^−1^ which indicates the O–H or N–H stretching groups, most probably from glucose and proteins. The band at 2,925.4 cm^−1^ can be assigned to C–H stretching. The absorption band at 1,584.5 cm^−1^ was due to the primary and secondary amides from the peptide bonds, which correspond to N–H bending. The absorption band in the region 1,300–1,050 cm^−1^ was due to the C–O bond, which is the characteristic peak for polysaccharides (Fig. 2). Absorption peaks were also observed in the region of 850–800 cm^−1^ which showed the involvement of cell surface sulfonate group. In the FT-IR spectrum of Cr(VI)-treated biomass, the band at 1,602 cm^−1^ was slightly shifted from the native biomass absorption peak of 1,584.5 cm^−1^. Shift of the band at 1,104.5 cm^−1^ from 1,117.3 and 1,078.8 cm^−1^ from 1,084 cm^−1^ were also observed.

### Identification of the heavy metal-resistant probiont LD22

**Fig. 3:**
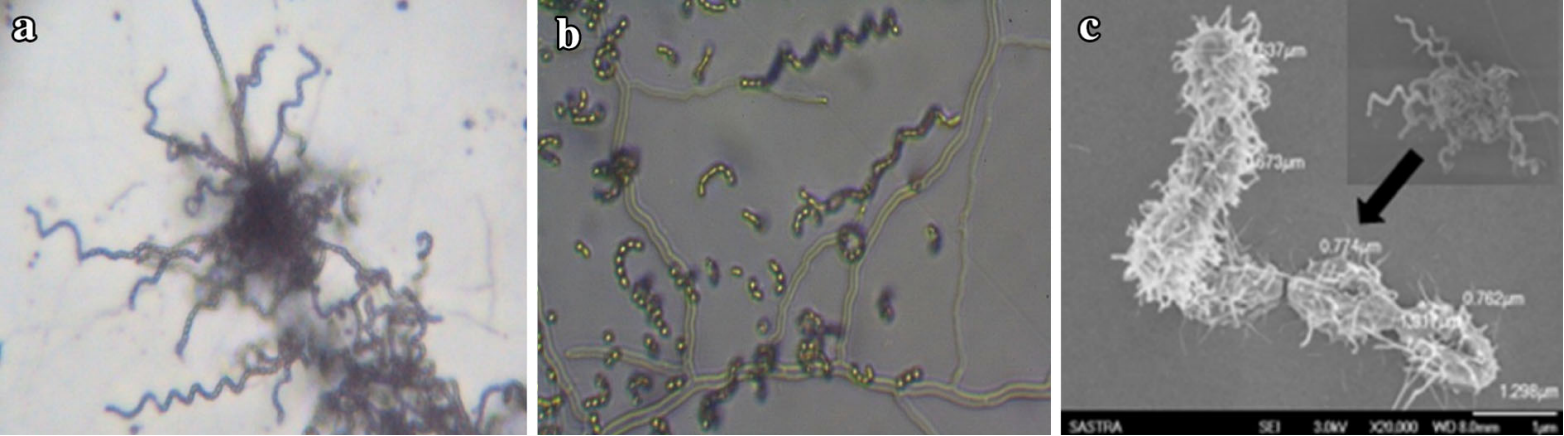
Micromorphology of isolate LD22 under light (**a**), phase contrast (**b**) and scanning electron (**c**) microscope

Microscopic observation revealed that the isolate LD22 was Gram positive and contained spore chains in the form of spirals. Thus, it was classified morphologically into section Spirales (S); mature spore chains generally contained 10–50 or more spores per chain. Conidial spores were spherical to ovoid in shape and had hairy surface (Fig. 3). Cultural characteristics study demonstrated that the isolate grew well on several media including ISP 1-7 and Bennett agar. In particular, aerial and vegetative hyphae were abundant in glycerol-asparagine agar (ISP5) and tyrosine agar (ISP7). The color of the aerial mycelium varied from gray, grayish white, white to yellow and the substrate mycelium ranged from gray, yellowish white, deep green to yellow. The size of the colony ranged from 5 to 10 mm and the consistency of the isolate was almost same in all the media, i.e., powdery in nature. The isolate produced light brown color-soluble pigment only in Bennett agar among the eight tested culture media. Moreover, melanin production was also absent, which was revealed by the absence of greenish-brown or black pigment on ISP media 6 and 7 (Supplement Table 2).

The nutritional characteristic study of the actinobacterial probiont LD22 showed that the isolate utilized all the sugars. In particular, good growth was observed in cellulose followed by xylose and galactose. The sugars sucrose, maltose and L-arabinose were less utilized by the isolate. Besides, growth of the isolate was significantly influenced by the amino acids arginine followed by tyrosine, valine, cysteine and glycine. However, the amino acids leucine and phenyl alanine less supported the growth of the isolate LD22. Excellent and moderate growth was observed in the pH range of 8–10 and 7, respectively, whereas poor growth was attained in the acidic pH range 5 and 6. In addition, the isolate tolerated up to 8 % of NaCl concentration and no growth was noticed at 10 % (Fig. S1).

The biochemical characteristics of LD22 displayed that the isolate was positive for catalase, oxidase, citrate and negative for indole, methyl red and voges–proskauer test. In the triple sugar iron agar slants, the formation of pink color in the butt and slant was observed which implies that the isolate did not metabolize all the three sugars such as lactose, glucose and sucrose. The absence of lifting up of medium and black precipitate specifies the negative result of gas and H_2_S production (Supplement Table 3). The isolate was positive for casein, urea, starch, lipid and gelatin hydrolysis.

**Fig. 4:**
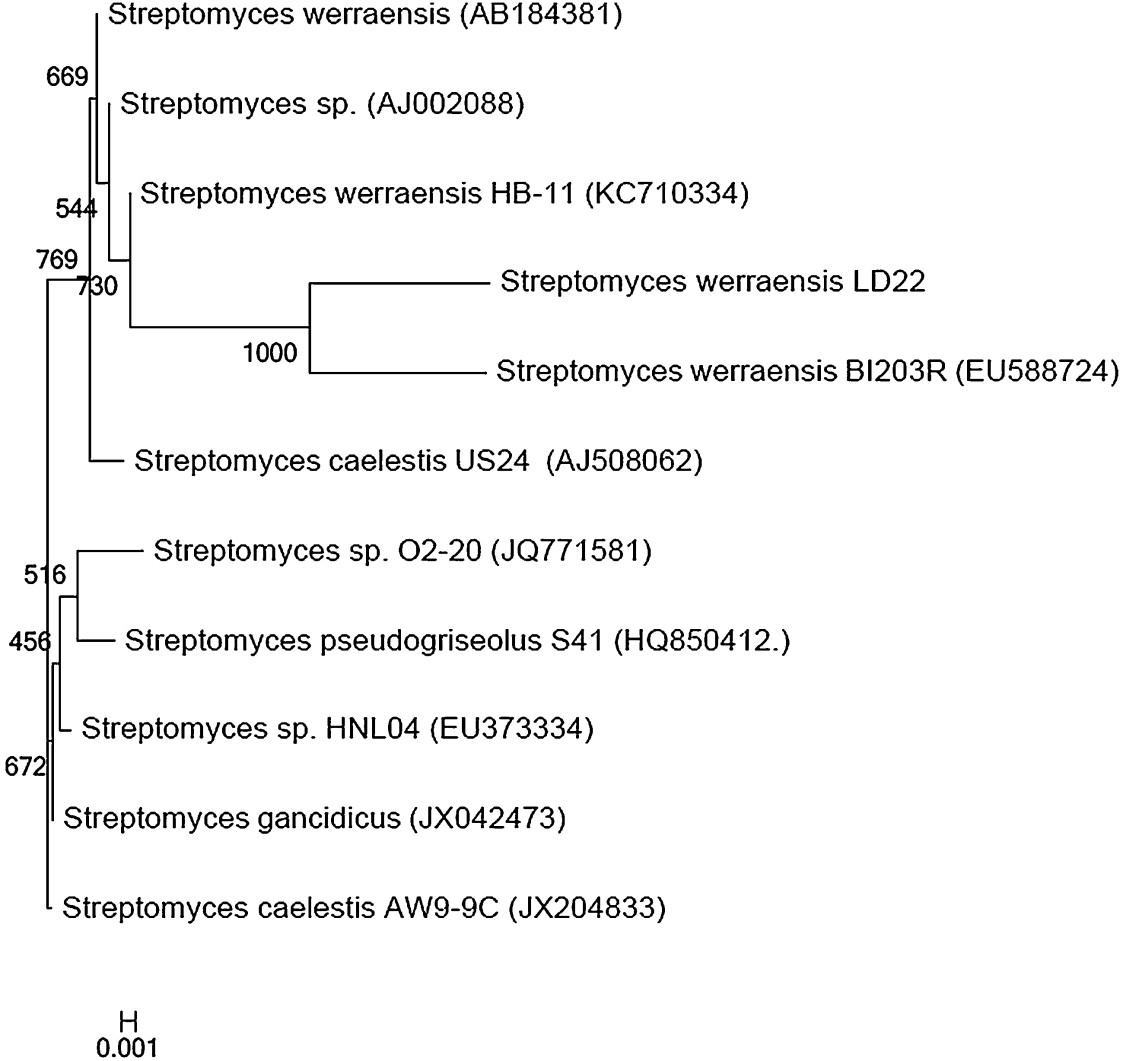
The phylogram showing the position of the isolate *Streptomyces werraensis* LD22 with other *Streptomyces* sp. based on 16S rRNA sequence. Bootstrap values (>50 %) are indicated at the relevant branching points. *Score bar* represents 1 nucleotide substitution per 1,000 nucleotides

PCR amplification of the genomic DNA with universal primers resulted in 1,078 bp amplicon. The 16S rRNA gene sequence thus obtained was subjected to BLAST similarity search with the NCBI database. The BLAST search result of the isolate LD22 showed 98 % similarity with *Streptomyces* *werraensis*. A phylogenetic tree was constructed based on neighbor-joining method (Fig. 4). Based on molecular phylogeny the isolate LD22 was designated as *S.* *werraensis* LD22 and the sequences were deposited in NCBI database with the accession number JX524481. The secondary structure of the 16S rRNA gene sequence of the potent actinobacterial isolate *S. werraensis* LD22 was predicted using the GeneBee tool. It showed 20 stems and 13 loops with an overall energy level of −95.4 kkal/mol (Fig. S2). The restriction sites present on the 16S rRNA gene of the isolate LD22 was analyzed using the NEBcutter program and it showed the site for various commercial and NEB (New England Biolabs) restriction enzymes. The number of restriction sites present on 16S rRNA gene sequences of *S. werraensis* LD22 was 55 and the GC and AT contents were 58 and 42 %, respectively (Fig. S3).

## Discussion

The heavy metals selected for the present study are toxic pollutants of the environment (soil, water, air, food) and potential threat to living organisms (humans, plants, animals, microbes) which have been evidenced by the accumulation of heavy metals such as Fe, Cu, Zn, Ni, Mn, Cd, Pb and Cr in soil (Li et al. 2006), animal feed (Okoye et al. 2011), chicken, quail (Abduljaleel et al. 2012), fishes (Amundsen et al. 1997; Wong et al. 2001), etc. Therefore, it is necessary to remove or detoxify these heavy metals to control the problem of bioaccumulation in living organisms, chiefly in livestock. In recent decades, conventional physical and chemical methods have been replaced by biosorption techniques due to their high expense, ineffectiveness and non-environment friendly nature. A vast array of biological materials, especially bacteria, yeasts and actinobacteria, was employed for heavy metal removal and recovery due to their good performance, low cost, large available quantities, non-harmful and non-pathogenic nature (Wang and Chen 2009). Besides, resident gut microflora that is symbiotic and helps the host in different ways also play a significant role in detoxification and elimination of harmful metals from humans and animals (Upreti et al. 2004). Thus, here also an attempt has been made to search for novel actinobacterial probionts with metal-resistant and metal-removal characteristics.

In the present study, all the tested probiotic actinobacterial isolates showed resistance toward K_2_Cr_2_O_7_, NiCl_2_, PbNO_3_, ZnCl_2_, CuSO_4_ and sensitivity to HgCl_2_ at 20 mg L^−1^ concentration in minimal agar medium. In addition, they exhibited multitolerance to heavy metals with the MTC values of **<**50–250 mg L^−1^. The results clearly revealed that the fecal actinobacterial isolates (gut actinobacteria) used in the present study had excellent heavy metal resistance properties, which might be due to adaptations developed during continuous exposure of heavy metals through metal-contaminated water and feed. Similarly, actinobacterial strains isolated from various sources also proved their ability to tolerate a variety of heavy metals at different concentrations (Abbas and Edwards 1989; Amoroso et al. 2000), which suggested that they acquired resistant properties probably due to frequent heavy metal exposure in the investigated regions, because adapted microbial populations are prone to show higher resistance to heavy metals as compared to populations of non-contaminated sites (Koushalshahi et al. 2012). Bhakta et al. (2012) reported the isolation of probiotic lactic acid bacterial cultures (LAB) from the environmental samples using MRS agar supplemented with 50 mg L^−1^ concentration of Cd and Pb. Among the 255 cadmium (109) and lead (146)-resistant colonies, 26 LAB exhibited remarkable probiotic and metal-removal characteristics (Bhakta et al. 2012). In the present study, minimal agar medium (MMA) was used for primary qualitative and semiquantitative metal resistance assays, because the supplemented metal does not form complexes with medium components and all the metals added were bioavailable (Amoroso et al. 2000).

Chromium is one of the highly toxic pollutants of the environment (e.g., water and subsurface soils), present in trivalent Cr(III) and hexavalent forms Cr(VI). Cr(VI) poses a greater risk due to its carcinogenic properties to living organisms, while Cr(III) is generally toxic to plants at very high concentrations and is less toxic or non-toxic to animals. Cr(VI) induces acute and chronic toxicity, neurotoxicity, dermatotoxicity, genotoxicity, immunotoxicity and general environmental toxicity (Bagchi et al. 2002). Moreover, in the current study almost all the tested isolates were highly tolerant to Cr(VI) at 250 mg L^−1^ concentration. Thus, the metal Cr(VI) was selected for the study of biosorption using the multimetal-tolerant isolate LD22.

The biosorption efficacy of the isolate LD22 for Cr(VI) was found to be maximum at 100 mg L^−1^ metal ion concentration (3 g L^−1^ of biomass dosage and pH 7.0). The higher biosorption ability with increased metal ion concentration could be attributed to higher probability of interaction between metal ions and biosorbents. However, the biosorption percentage was considerably decreased with the increased concentration of Cr(VI) which might be due to the saturation of metals in biosorption. Thus, the results indicate that the initial metal concentration plays a significant role in metal biosorption. The results of this experiment were in agreement with the report of Saurav and Kannabiran (2011a, b). Their study revealed that the optimum biosorption efficacy of Cr(VI) was obtained for *Streptomyces* VITSVK9 spp. at 100 mg L^−1^ metal ion concentration, pH 7.0 and biosorbent dosage of 3 g L^−1^. Earlier studies have indicated that the initial metal ion concentration (Saurav and Kannabiran 2011b) biosorbent dose (Yao et al. 2009; Acharya et al. 2009) and pH (Aksu et al. 1991; Donmez et al. 1999) were the important parameters affecting biosorption capacity as well as removal efficiency.

The determination of chromium uptake was further supported by FT-IR spectroscopy which analyzed the possible interactions between the functional groups of LD22 biomass with chromium. The FT-IR spectrum of native culture biomass revealed the chemical interactions between the hydroxyl (O–H), amine (N–H) and carboxyl (C–O) groups of the biomass and the metal ions. The FT-IR spectrum of Cr(VI)-treated biomass differed from the native biomass of LD22 in its absorption peaks, which might be due to the secretion of high molecular mass polymers that can either be released into the environment or remain attached to cell surfaces. Similar findings were reported for chromium-treated *Acinetobacter* sp. B9 (Bhattacharya and Gupta 2013), *A. haemolyticus* (Pei et al. 2009) and *Streptomyces* sp. VITSVK9 (Saurav and Kannabiran 2011b). The cell wall of the *Streptomyces* sp. VITSVK9 biomass likely contains several functional groups which can play an important role in the biosorption of metal ions. In general, microbial cell walls are known to be rich in polysaccharide and glycoproteins such as glucans, chitin, mannans and phospho-mannans. The metal-binding properties of Gram positive bacteria, such as actinomycetes (*Streptomyces*), are largely due to the existence of specific anionic polymers in the cell wall structures, which consist mainly of peptidoglycan, teichoic or teichuronic acids. It is particularly the cell wall structures of certain algae, fungi and bacteria, which have been found to be responsible for metal ion interaction (Volesky 1990).

The morphology of the isolate LD22 was similar to that described by Wallhausser et al. (1964) and Muharram et al. (2013), which clearly indicates that the isolate under investigation belongs to the genus *Streptomyces.* In the present study, the isolate showed spiral spore morphology which agreed well with the descriptions of *S. werraensis* (Shirling and Gottlieb 1972; Wallhausser et al. 1964). Inoue et al. (1975) also reported the same organism with a different type of spore morphology manifested as Rectiflexibiles or Spirales. It did not show so much deviation from the description in the ISP. However, the cultural characteristic of our isolate was mostly similar to the report of Inoue et al. (1975), particularly in powdery consistency, absence of soluble and melanin production, color of aerial and substrate mycelium. Although the isolate LD22 utilized all the tested carbon and nitrogen sources, growth was significantly high in cellulose and arginine which revealed that the isolate utilized them as sole carbon and nitrogen source for its growth. Excellent growth in high pH (8–10) demonstrated that the isolate was alkalophilic in nature. The growth in the presence of salt concentration displayed it was abundant in low (2 %) NaCl concentration, but slowly reduced in high (10 %) NaCl concentration. The isolate also showed various biochemical activities such as utilization of citrate, absence of carbohydrate fermentation and the ability to produce different enzymes such as amylase, urease, protease, lipase, gelatinase, catalase and cytochrome oxidase.

The phylogenetic relationship revealed that the isolate LD22 was strongly related with other *S.* *werraensis*; thus the isolate LD22 was justifiably identified as *S.* *werraensis* LD22. The 16S rRNA sequence of the same isolate was deposited in NCBI database with the accession number JX524481. Several researchers have reported the utility of 16S rRNA sequence comparison/evaluation as a tool to confirm the identity of actinomycetes (Dhanasekaran et al. 2012; Saha et al. 2013).

In conclusion, the present investigation revealed that the probiotic actinobacterial isolates have the ability to resist hazardous heavy metals, such as K_2_Cr_2_O_7_, NiCl_2_, CuSO_4_, PbNO_3_ and ZnCl_2_, in humans and animals. Among the 20 probiotic actinobacterial cultures, the probiont LD22 demonstrated multiple heavy metal tolerance with the maximum Cr(VI) biosorption efficacy of 82.3 % at 100 mg L^−1^. On the basis of phenotypic, physiological, biochemical and molecular characteristics, the potent probiotic Cr(VI) biosorbent LD22 was identified as *S.* *werraensis* LD22 which could be used to protect livestock from the problems of heavy metal bioaccumulation and biomagnifications with other beneficial health effects.

## Electronic supplementary material

Below is the link to the electronic supplementary material.

Supplementary material 1 (DOC 732 kb)

Supplementary material 2 (DOC 35 kb)
