# Supplementary material for: Chromium [Cr(VI)] biosorption property of the newly isolated actinobacterial probiont Streptomyces werraensis LD22
Source: 3 Biotech. 2014 Jul 14;5(4):423–32. doi: 10.1007/s13205-014-0237-6 (PMC4522735; doi:10.1007/s13205-014-0237-6)
Supplement: Supplementary file 1 — Supplementary material 1 (DOC 732 kb) [file 13205_2014_237_MOESM1_ESM.doc]

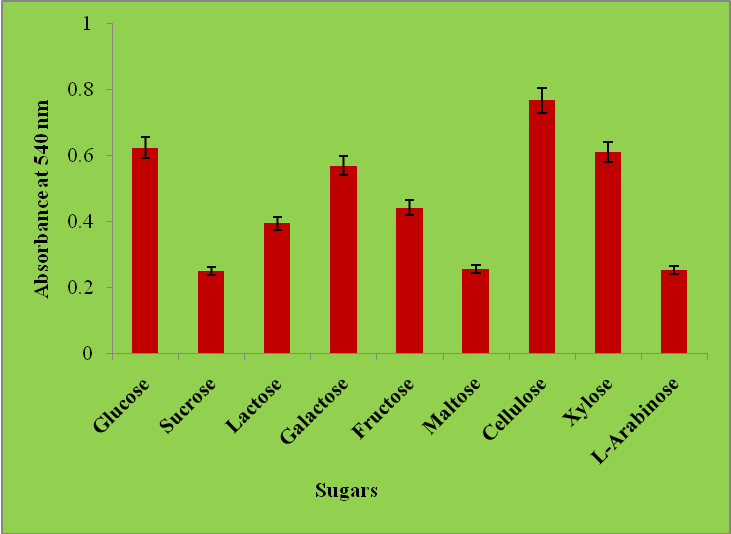


**a.** Utilization of carbon sources by the isolate LD22

**
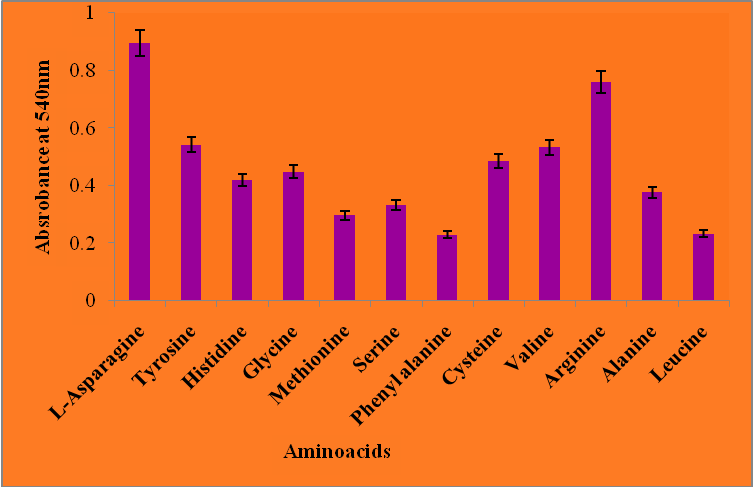
**

**b.** Utilization of nitrogen sources by the isolate LD22

**Fig. S1** Physiological characterization of isolate LD22


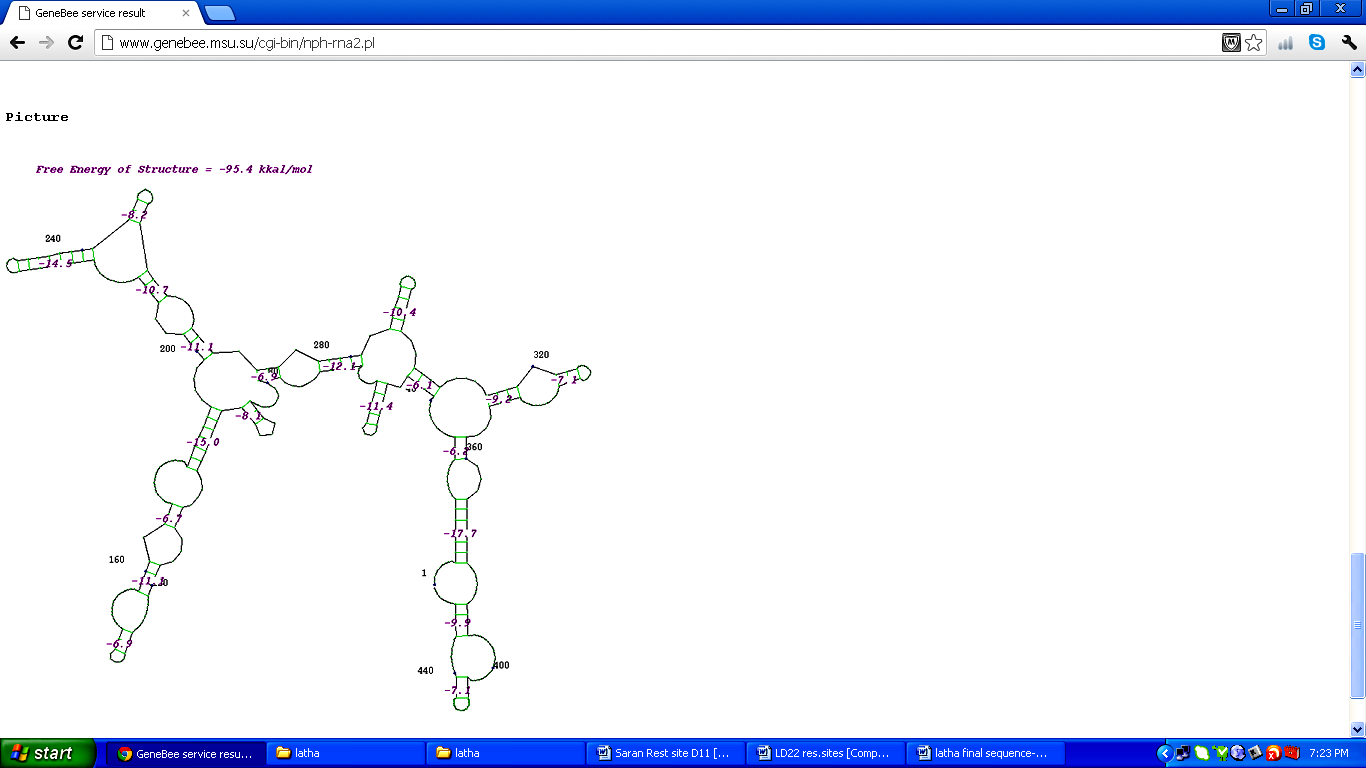


**Fig. S2** Secondary structure of 16S rRNA gene sequence of

*Streptomyces* *werraensis* LD22 isolate using Genebee software

| 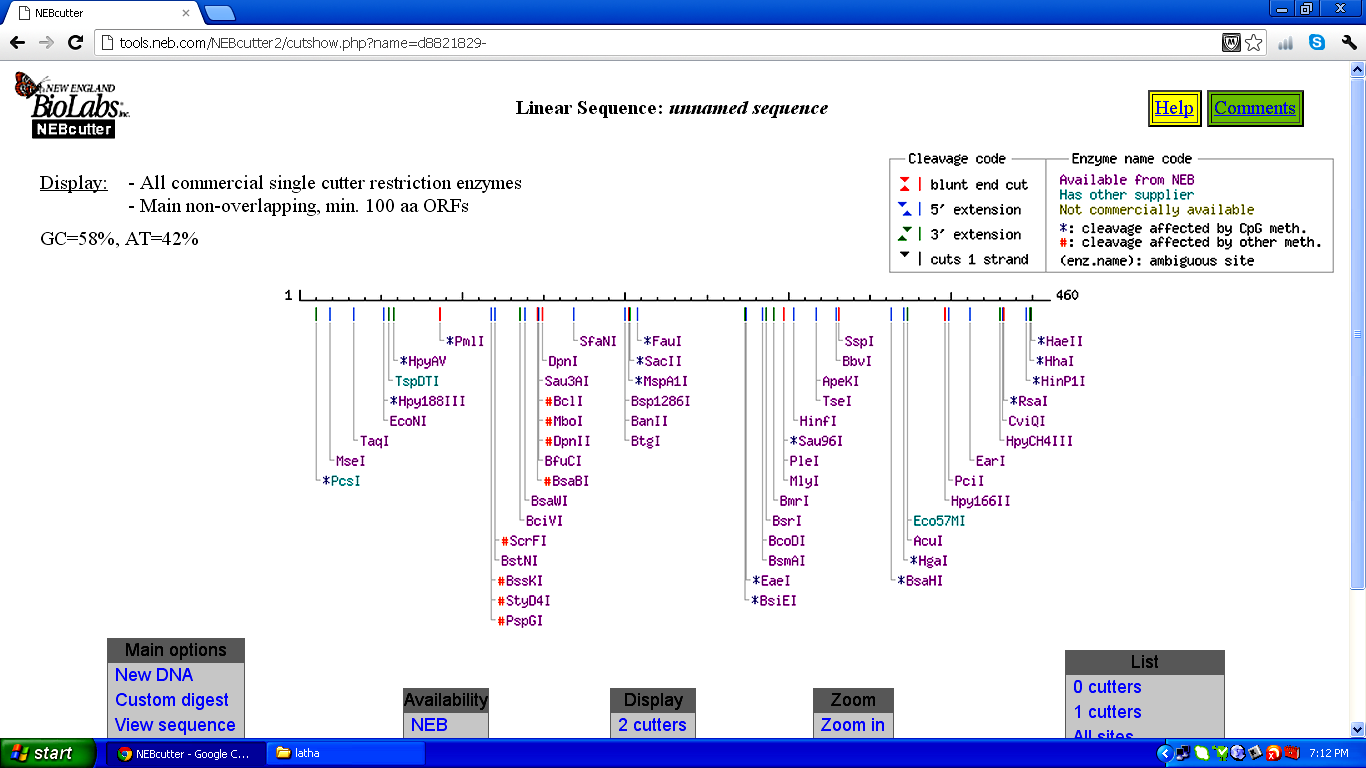 | 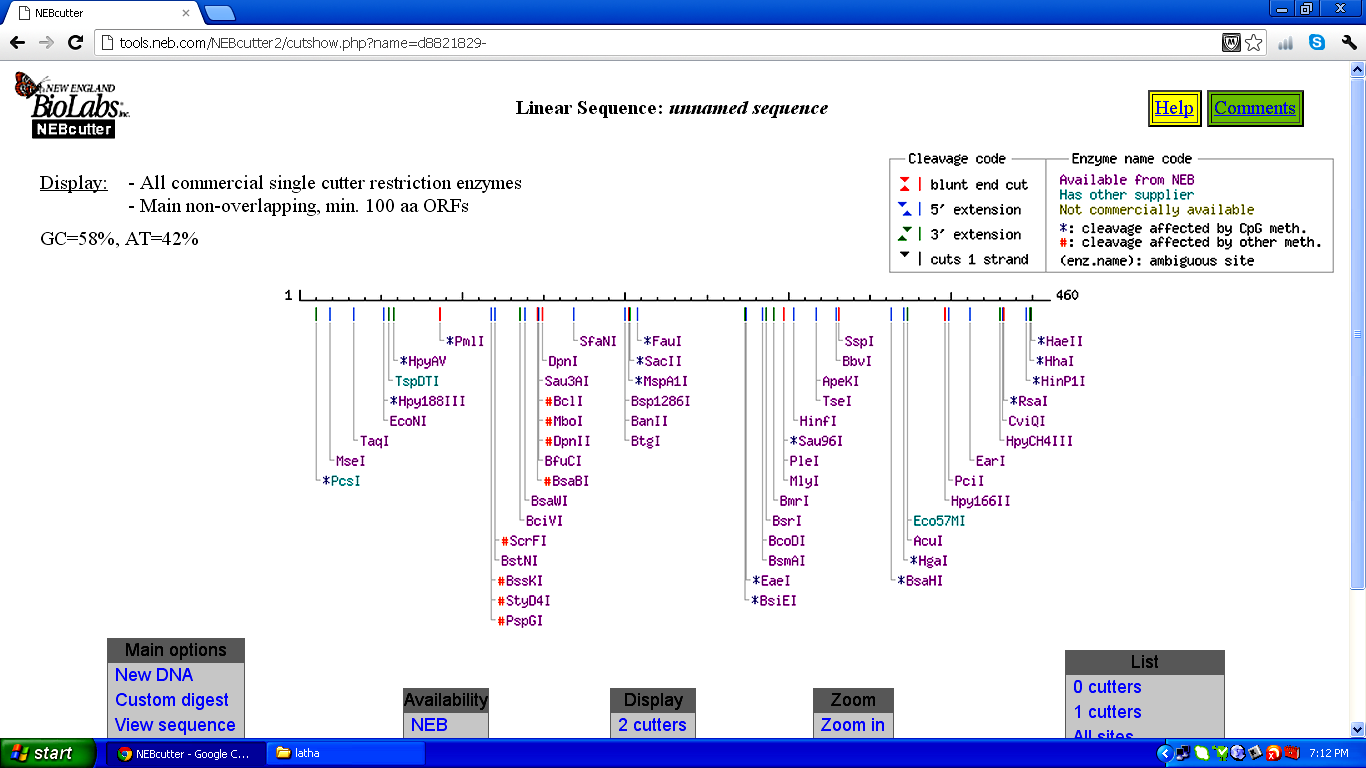 |
| --- | --- |
| **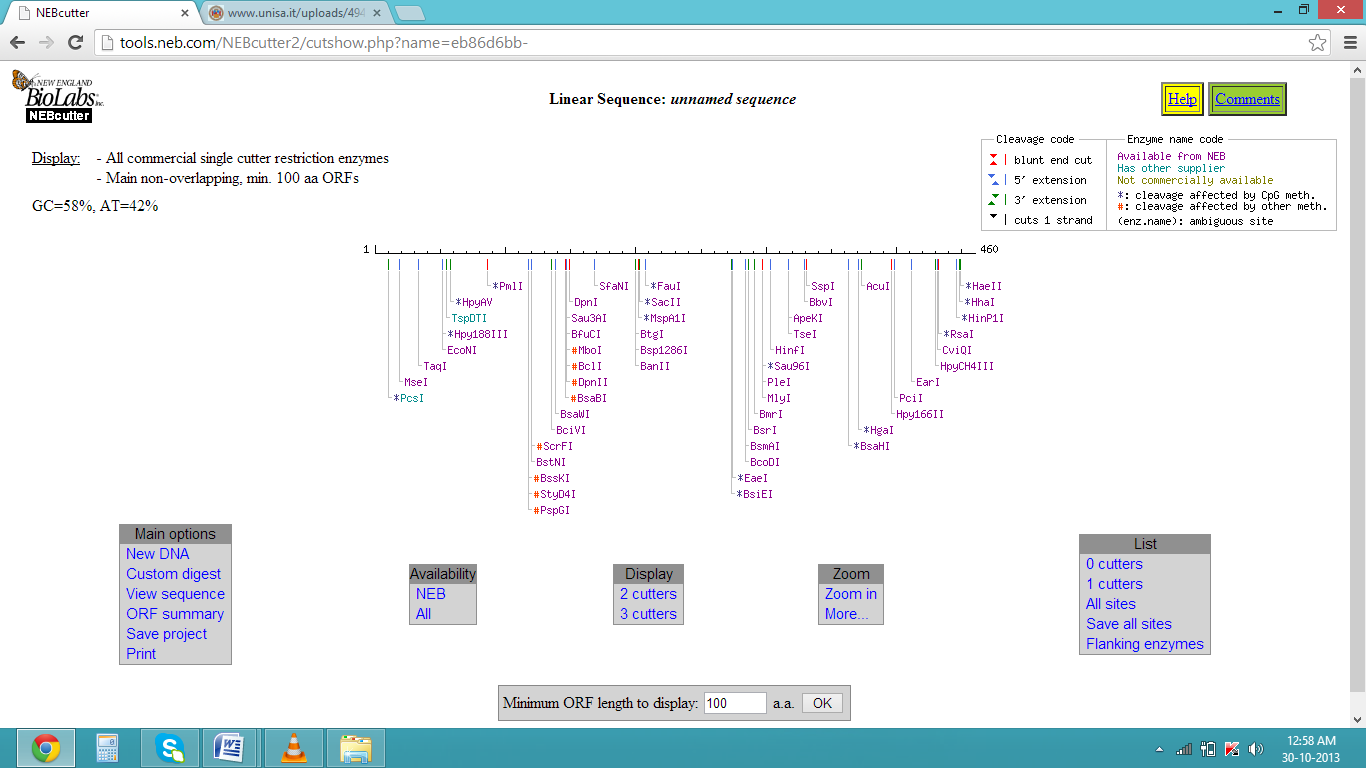** | |

**Fig. S3** Restriction site analysis of 16S rRNA gene sequence of

*Streptomyces* *werraensis* LD22 isolate using NEB Cutter program
