# Supplementary material for: Chromium [Cr(VI)] biosorption property of the newly isolated actinobacterial probiont Streptomyces werraensis LD22
Source: 3 Biotech. 2014 Jul 14;5(4):423–32. doi: 10.1007/s13205-014-0237-6 (PMC4522735; doi:10.1007/s13205-014-0237-6)
Supplement: Supplementary file 2 — Supplementary material 2 (DOC 35 kb) [file 13205_2014_237_MOESM2_ESM.doc]

**Table 2** Cultural characteristics of the heavy metal resistant probiont LD22

| **Media** | **LD22** |
| --- | --- |
| **1. Tryptone yeast extract agar (ISP1)** | |
| Colony size (mm) | 10 |
| Consistency | Powdery |
| Aerial mycelium | Grey |
| Substrate mycelium | Dull grey |
| Growth rate | Moderate |
| Pigment production | **-** |
| **2. Yeast extract malt extract agar (ISP2)** | |
| Colony size (mm) | 5 |
| Consistency | Powdery |
| Aerial mycelium | Greyish white |
| Substrate mycelium | Yellowish grey |
| Growth rate | Moderate |
| Pigment production | - |
| **3. Oat meal agar (ISP3)** | |
| Colony size (mm) | 4 |
| Consistency | Powdery |
| Aerial mycelium | Dark grey to white |
| Substrate mycelium | Grey |
| Growth rate | Moderate |
| Pigment production | **-** |
| **4. Inorganic salt starch agar (ISP4)** | |
| Colony size (mm) | 5 |
| Consistency | Powdery |
| Aerial mycelium | Ash white |
| Substrate mycelium | Greenish white |
| Growth rate | Moderate |
| Pigment production | - |

| **Media** | **LD22** |
| --- | --- |
| **5. Glycerol - asparagine agar (ISP5)** | |
| Colony size (mm) | 5 |
| Consistency | Powdery |
| Aerial mycelium | White |
| Substrate mycelium | Yellowish white |
| Growth rate | Abundant |
| Pigment production | - |
| **6. Peptone yeast extract iron agar (ISP6)** | |
| Colony size (mm) | 5 |
| Consistency | Powdery |
| Aerial mycelium | Yellow |
| Substrate mycelium | Yellowish white |
| Growth rate | Moderate |
| Pigment production | - |
| **7. Tyrosine agar (ISP7)** | |
| Colony size (mm) | 5 |
| Consistency | Powdery |
| Aerial mycelium | Greyish white |
| Substrate mycelium | Deep green |
| Growth rate | Abundant |
| Pigment production | - |
| **8. Bennett’s agar** | |
| Colony size (mm) | 7 |
| Consistency | Powdery |
| Aerial mycelium | Greyish white |
| Substrate mycelium | Deep yellow |
| Growth rate | Moderate |
| Pigment production | Light brown |

**Table 3** Characteristics of the heavy metal resistant probiont LD22

| **S. No.** | **Name of the test** | **LD22** |
| --- | --- | --- |
| 1. | IMViC | **---+** |
| 2. | Triple sugar iron agar | **K/K** |
| 3. | H_2_S production | **-** |
| 4. | Gas Production | **-** |
| 5. | Catalase | + |
| 6. | Oxidase | + |
| 7. | **Enzyme activities** | |
|  | Urea | + |
|  | Casein | + |
|  | Starch | + |
|  | Lipid | + |
|  | Gelatin | + |
| 8. | **Effect of pH** | |
|  | 5 | + |
|  | 6 | ++ |
|  | 7 | +++ |
|  | 8 | ++++ |
|  | 9 | ++++ |
|  | 10 | ++++ |
| 9. | **Effect of NaCl concentration** | |
|  | 2% | ++++ |
|  | 4% | +++ |
|  | 6% | +++ |
|  | 8% | + |
|  | 10% | - |

IMViC – Indole, Methyl red, Voges-proskauer, Citrate

K/K – Alkaline/Alkaline, (+) Positive, (-) Negative

(- ) No growth, **(+**,***++*)** Poor growth, (*+++*) Moderate growth, (***++++*)** Excellent growth
